# A gene catalogue for post-diapause development of an anhydrobiotic arthropod *Artemia franciscana*

**DOI:** 10.1186/1471-2164-10-52

**Published:** 2009-01-27

**Authors:** Wie-Hua Chen, Xiaomeng Ge, Weiwei Wang, Jun Yu, Songnian Hu

**Affiliations:** 1 CAS Key Laboratory of Genome Sciences and Information, Beijing Institute of Genomics, Chinese Academy of Sciences, Beijing, 100029, PR China; 2 European Molecular Biology Laboratory (EMBL), Meyerhofstrasse 1, 69117 Heidelberg, Germany

## Abstract

**Background:**

Diapause is a reversible state of developmental suspension and found among diverse taxa, from plants to animals, including marsupials and some other mammals. Although previous work has accumulated ample data, the molecular mechanism underlying diapause and reactivation from it remain elusive.

**Results:**

Using *Artemia franciscana*, a model organism to study the development of post-diapause embryos in Arthropod, we sequenced random clones up to a total of 28,039 ESTs from four cDNA libraries made from dehydrated cysts and three time points after rehydration/reactivation, which were assembled into 8,018 unigene clusters. We identified 324 differentially-expressed genes (DEGs, *P *< 0.05) based on pairwise comparisons of the four cDNA libraries. We identified a group of genes that are involved in an anti-water-deficit system, including proteases, protease inhibitors, heat shock proteins, and several novel members of the late embryogenesis abundant (LEA) protein family. In addition, we classified most of the up-regulated genes after cyst reactivation into metabolism, biosynthesis, transcription, and translation, and this result is consistent with the rapid development of the embryo. Some of the specific expressions of DEGs were confirmed experimentally based on quantitative real-time PCR.

**Conclusion:**

We found that the first 5-hour period after rehydration is most important for embryonic reactivation of *Artemia*. As the total number of expressed genes increases significantly, the majority of DEGs were also identified in this period, including a group of water-deficient-induced genes. A group of genes with similar functions have been described in plant seeds; for instance, one of the novel LEA members shares ~70% amino-acid identity with an *Arabidopsis *EM (embryonic abundant) protein, the closest animal relative to plant LEA families identified thus far. Our findings also suggested that not only nutrition, but also mRNAs are produced and stored during cyst formation to support rapid development after reactivation.

## Background

Diapause, also known as discontinuous development, is a reversible state of developmental suspension often promoted by seasonal environmental adversity cues. It is a strategy that ensures successful species survival through timing the post-diapause development for favorable environmental conditions. This protective mechanism is widely distributed among different taxa, including plants, insects, vertebrates, and even some mammals and it occurs at different developmental stages, such as embryonic, larval, pupal, or adult stages, often varying from species to species.

*Artemia *is a group of small ancient crustaceans living in saline waters. Induced by signals from forthcoming seasonal adversities (not by the adversities themselves), *Artemia *produces shelled embryos (cysts) that suspend development and metabolism at the gastrula stage, which contain around 4,000 cells [1,2]. These cysts are in a state of diapause, a physiological dormancy with specific releasing conditions. The primary important feature of diapause is that once initiated, it can only be released by certain stimuli; until then, the cysts wouldn't continue to develop even putting them into favorable environments. This feature is essential in distinguishing diapause as a different phenomenon from other forms of dormancy such as hibernation and quiescence [3]. In *Artemia*, the diapause could be broken by dehydration (often complete dehydration is required) and is followed by a state of quiescence, from which the cysts can begin direct development, should conditions change to become more favorable. The diapause-broken was often considered as a mechanical process instead of physiological transition, because a) the time of the dehydration process doesn't matter to the hatching rate; b) multiple-round of dehydration- rehydration is sometime necessary to diapause-break and c) little physiological and biological changes were observed [4]. These dehydrated cysts are able to survive years even decades, with little signs of metabolism and energy consumption, but remain viable [5-7]. Once reactivated in favorable environments, they resume development and give rise to free-swimming larva within 24 hours. The developing of embryonic development of *Artemia *is generally divided into three stages, namely pre-diapause, diapause and post-diapause [4].

Physiologically and biologically, the encysted embryos resemble plant seeds in many aspects. For example, both contain embryos that develop to certain stages and undergo complete dehydration without any obvious loss of viability even stored for years. Recent studies also revealed that certain molecules such as heat shock proteins are significantly enriched in both cysts and plant seeds.

As an initial step to address the molecular mechanisms of diapause, we first of all constructed a cDNA library using dehydrated cysts that were in a state of quiescence and likely to contain genes important to the maintenance of diapause, since no physiological changes observed during the transition. Then we analyzed its gene expression profiles and compared with that of cDNA libraries that were derived from three time points of subsequent development after rehydration. Expression sequence tags (ESTs) were used to get quick access to gene sequences and expression information of the developing *Artemia*. We obtained ~30,000 high-quality ESTs from the four cDNA libraries in approximately equal numbers from each, and clustered them into 8,018 unigenes after sequence assembly and annotation. We also validated a few gene expression profiles using quantitative real-time PCR. All EST sequences were deposited into NCBI's dbEST under accessions from ES492186 to ES529129.

## Results and discussions

### Library construction and sequence assembly

We constructed four directional, non-normalized cDNA libraries for the brine shrimp, *Artemia franciscana*, using RNA samples extracted from dehydrated cysts (post-diapaused cysts) as well as three developmental stages at 5, 10, 15 hours after rehydration and named them as AfD0, AfR5, AfR10 and AfR15, respectively. We randomly sequenced ~10,000 clones from the 5'-end. After eliminating host gene contaminations, trimming vector and low-quality (< 100 bp in length) sequences, we obtained 28,039 high-quality EST sequences with an average length of 471 bp. The sequences were clustered into 8,018 unigenes that contain 1,951 contigs and 6,067 singlets (Table 1). The largest cluster has 601 ESTs.

**Table 1:** Summary of cDNA libraries

Library	Clone	Acquired Sequence	High-Quality Sequence	Assembled Unigene
AfD0	9,002	8,130	7,567	1,698
AfR5	8,178	7,453	6,832	3,146
AfR10	8,046	7,543	6,901	2,787
AfR15	7,988	7,473	6,739	3,006
Total	33,214	30,599	28,039	8,018

### Annotation of *Artemia *unigenes

**Figure 1:**
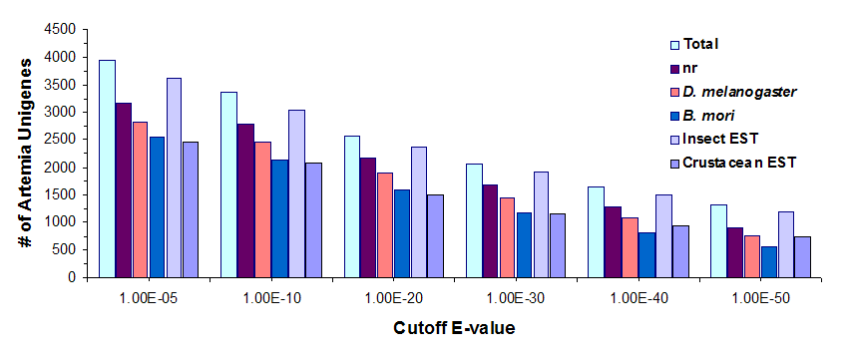
**Sequence comparisons with other relevant species**. Numbers of *Artemia *unigenes significantly matched to sequences in NCBI's non-redundant protein (nr) database, *Drosophila melanogaster *protein database (release 4.3), silkworm (*Bombyx mori*) BGF protein database, and other available EST sequences of *Insecta *and *Crustacea *from dbEST.

**Figure 2:**
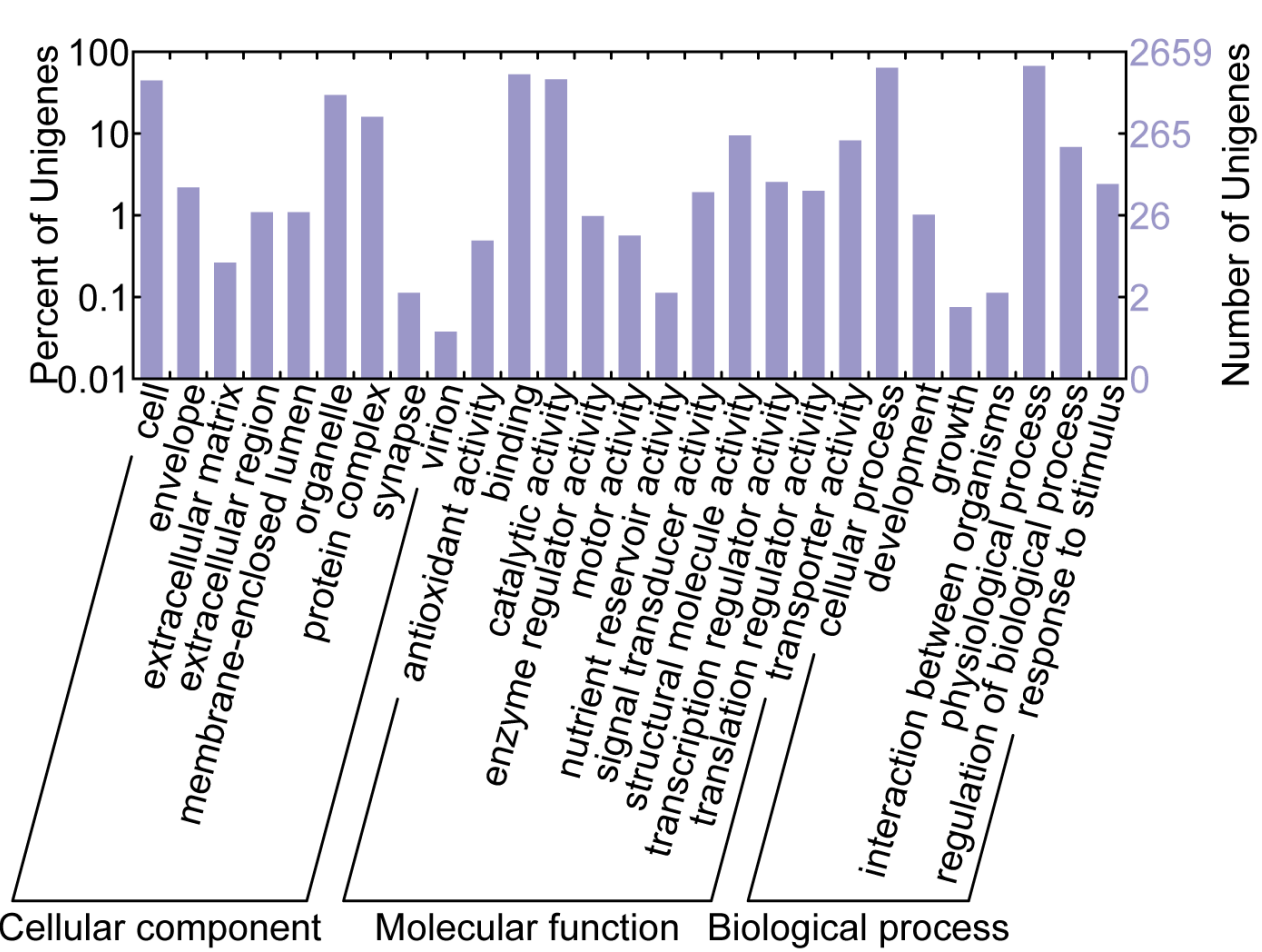
**Functional categorization of Artemia unigenes**. Gene Ontology classifications of *Artemia *unigenes with significant hits in Uniprot.

We annotated the *Artemia *unigenes by comparing to protein sequences collected from public databases (Figure 1), and 3,953 unigenes (49.3%) showed significant sequence similarities (e-value < 1e-5) to known genes. To determine whether the low sequence similarities of the remaining unigenes were attributable to short open reading frames (ORFs), we used *getorf *[8] to examine their putative ORFs. The results indicated that only 2.6% of the non-matching unigenes contained ORFs less than 30 amino acids. We noted that an even smaller portion (3,214) of the unigenes had shown significant similarities with the pre-release genome annotation FM3 (filtered models release 3) of *Daphnia*, a close relative of *Artemia *that shared many physiological similarities, indicating high genetic diversities among Crustacean. An overview of functional categories (Gene Ontology, GO) of annotated unigenes is shown in Figure 2.

### Expression profiles of *Artemia *unigenes in four cDNA libraries

We obtained 1,698 unigenes from AfD0 that derived from dehydrated cysts where metabolism and development are nearly suspended. We obtained significantly more unigenes from the other three cDNA libraries: 3,146, 2,787, and 3,006 from AfR5, AfR10, and AfR15, respectively (Table 1). We also carried out pair-wise comparisons for expression profiles among the libraries. For example, we identified 362 unigenes expressed in AfD0 and AfR5, 560 in AfR5 and AfR10, and 558 in AfR10 and AfR15. Only 194 unigenes were found universally expressed and nearly 50% unigenes were found unique to each library. Expression abundances of the unigenes correlate to their distributions in four libraries. For example, universally presented genes were highly expressed, with a mean cluster size of 46.7; the cluster sizes of those unique unigenes strongly biased toward very low values (around one EST per unigene). Genes expressed in two libraries showed in-between abundances with a mean cluster size of 21.2.

**Figure 3:**
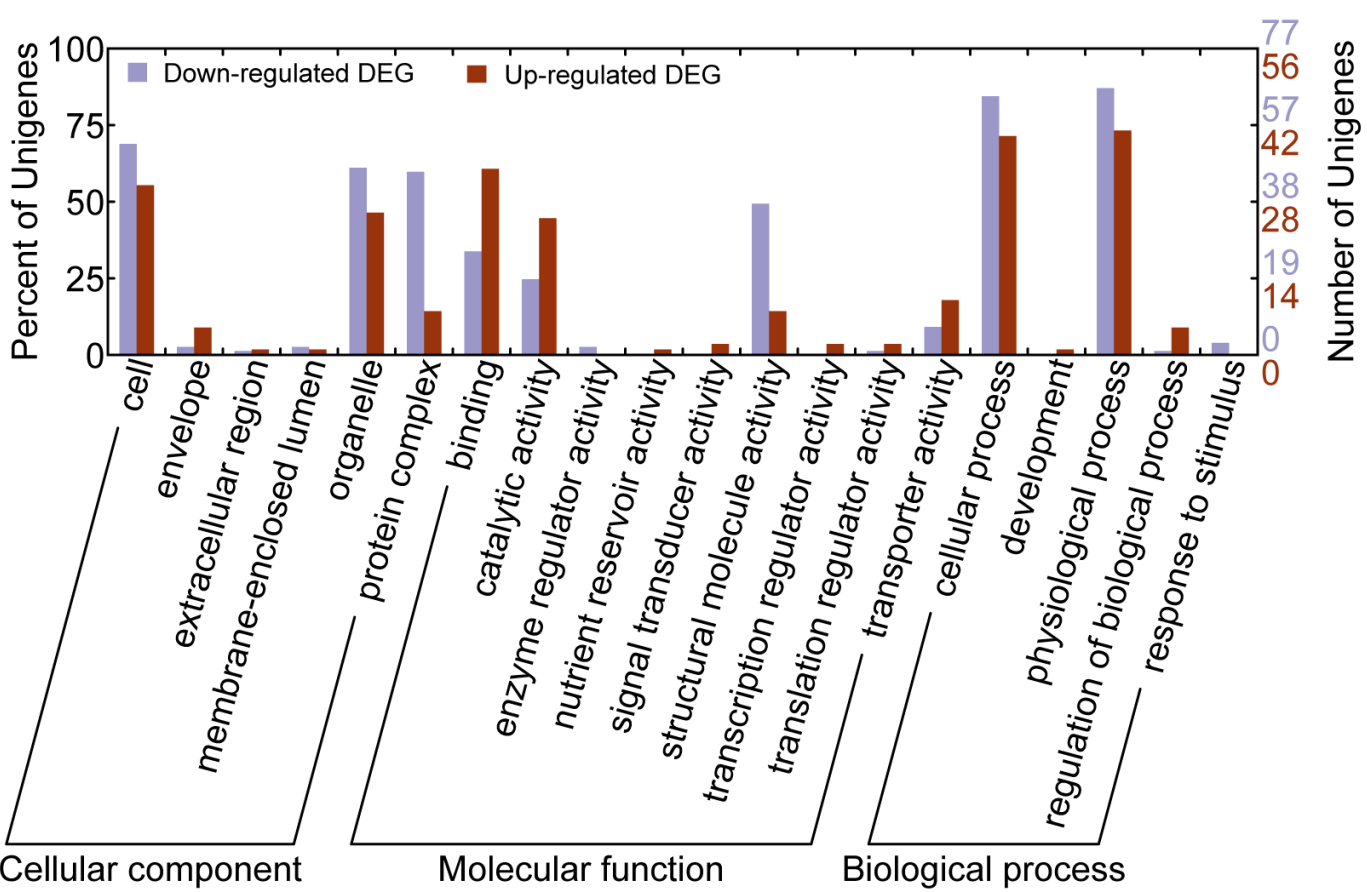
**GO classification of differentially-expressed genes (DEGs) between diapause cysts (AfD0) and developing Artemia in early developmental stage (AfR5)**.

To identify differentially expressed genes (DEGs) that are statistically significant, we adopted the general Chi-square algorithm to test expression profiles as previous studies suggested it was applicable and reliable for EST analysis [9]. We identified 324 DEGs with p-value < 0.05, including 279 between AfD0 and AfR5, 64 between AfR5 and AfR10, and 50 between AfR10 and AfR15. This algorithm masked most of the unigenes that were unique to any of the cDNA libraries as only 21 of them were assigned as DEGs. Our results suggested that a major change occurred in the first five hours after reactivation. We annotated 56 up-regulated and 77 down-regulated genes from 133 DEGs between AfD0 and AfR5 (47.3%, out of 279 DEGs). We assigned one or more GO terms to all these 133 genes by an annotation transfer process (see Materials and Methods). As shown in Figure 3, GO categories of cell, organelle, protein complex, structural molecular activity, cellular process, and physiological process are most abundant among down-regulated DEGs, and protein complex, enzyme regulator activity, and response to stimulus are abundant among up-regulated DEGs. A full list of DEGs is shown in Additional File 1.

### Down-regulated and up-regulated DEGs after reactivation

We identified 142 down-regulated genes after reactivation and annotated 77 of them. We grouped cysts-enriched transcripts into several categories based on their putative biological functions, such as stored in cysts and utilized after reactivation or involved in anti-desiccation and diapause. We also look into ribosomal proteins as they are stored in cysts and used after reactivation to facilitate quick physiological and biological transition from the quiescence to the active states [10], and identified 37 ribosomal protein coding genes as down-regulated DEGs. In addition, we noticed a cathepsin-like cysteine proteinase (CLAP) that is stored in cysts but activated after rehydration and involved in yolk utilization in *Artemia *embryos [11]. However, other studies shown that proteinases were involved in desiccation-tolerance and the accumulation of proteases was often correlated with increased amount of aggregated/denatured proteins induced by water-loss [12]. As proteases are often multi-functional, our efforts to assign them into function-related groups became difficult sometimes. For example, we identified another down-regulated HAD-like protease (haloacid dehalogenase-like hydrolase) but categorized it as water-loss induced genes.

Water-deficit induces the expression of protease inhibitors and chaperones and serve as counteracting mechanisms to prevent protein degradation. Among down-regulated DEGs, we identified a cysteine protease inhibitor (Cystatin B) that binds tightly to and inhibit papain, cathepsin B and lysosomal cathepsin L [13] and several chaperone genes, including late embryogenesis abundant proteins (LEAs), a small heat shock protein p26, and HSP70. As classic chaperone, p26 was previous found abundantly expressed in encysted embryos of *Artemia *and it was suggested to protect proteins from irreversible denaturation in an energy-independent manner [14] and cells against oxidative damage [15]. In stressed cells, HSP70 and p26 move to the nucleus upon stresses and played a role in stabilization of nuclear matrix proteins [16].

**Figure 4:**
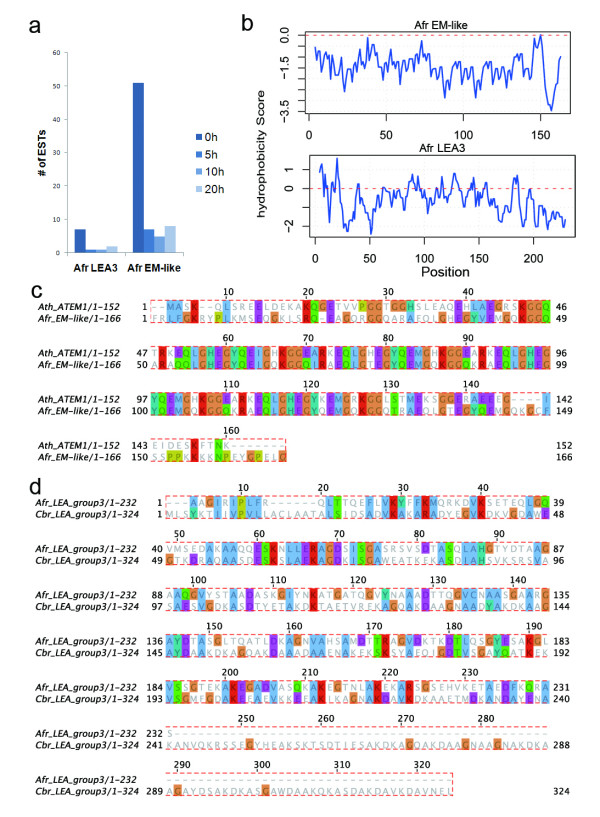
**Expression abundances and sequence features of two novel LEA genes in Artemia**.

LEAs are a group of new and non-classic chaperones. They were originally identified in plant seeds and recently in animals. Considerable evidence suggested that LEA proteins were involved in desiccation resistance, by means of water retention, sequestration of ions, direct protection of other proteins or membranes [12,17], although the exact molecular mechanisms how these protein function in the cell remain elusive. From the 8,018 unigenes, we identified three LEA genes, including two novel members, Afr_EM-like and Afr_LEA3, and both were down-regulated after reactivation. As shown in Figure 4a, their expression patterns were consistent with their putative functions in desiccation tolerance. Both encode strongly hydrophilic proteins (Figure 4b). The novel Afr_EM-like protein contains a LEA_5 domain (PF00477) with an e-value of 8.6e-07 in PFAM database, and showed 65% amino acid identity and 74% similarity to the Em (embryonic abundant, GenBank accession NP_190749, Figure 4c) protein of *Arabidopsis thaliana*, which is the closest LEA relative in plants identified so far. The other novel Afr_LEA3 gene coded for a protein containing a LEA_3 domain, and had best match with LEA3 protein (GenBank Accession XP_001675059) of *Caenorhabditis briggsae *with relatively low similarity (27% identities and 45% similarity, Figure 4d). Two LEA genes, AfrLEA1 and AfrLEA2, were reported previously and showed to have a differential expression between post-diapaused embryos and free-swing larva of *Artemia franciscana *based on quantitative real-time PCR (qRT-PCR) [18]. However, only AfrLEA2 was identified in our cDNA library, which matched to a single EST and did not show differential expression among our cDNA libraries. The discrepancy was most likely attributable to different technique routes adopted in the two studies: qRT-PCR is much more sensitive to low abundant genes, while cDNA cloning can only capture abundantly expressed transcripts. We also identified many other genes that are significantly enriched in dehydrated embryos, including SEC-like, elongation factor 2 (EF-2), larval cuticle and chitin binding proteins.

In a previous study, Qiu Z. *et al *identified ~85 genes enriched in the diapause-destined embryos of *Artemia franciscana *by using a subtractive cDNA library [19]. We found only nine down-regulated DEGs that are shred between the two datasets, including those of p26, superoxide dismutase, and several ribosomal proteins (Additional file 2). Since only less than 300 cDNA clones were sequenced in the study and our sampling depth is also rather shallow, we did not anticipate much overlapping data.

Through our annotation pipeline, we were able to annotate 56 of 137 up-regulated DEGs in four cDNA libraries. From them, we identified several functionally related groups, including those involved in (1) metabolism and ATP generation, (2) cell growth and cell division, (3) transcription and translation, and (4) folding of newly translated proteins. The increasing expression of these genes was consistent with the rapid development after reactivation of *Artemia *cysts, which usually gives rise to free-swimming larva within 24 hours.

### Validation of EST expressions by qRT-PCR

To validate our EST sequencing results, we performed quantitative RT-PCR) for nine selected genes that showed differential expression, in which six down-regulated and three up-regulated DEGs. The down-regulated genes are three stress-tolerance genes, p26, HSP70 and LEA, two proteases, CLAP and HAD-like, and a protease inhibitor, Cystatin B inhibitor-like; three up-regulated genes are cell-cycle regulator CDC48, chaperonin-containing TCP that help fold newly translated proteins, and a UCP2. Since all these DEGs showed differential expression between 0 h and 5 h, we only performed qRT-PCR experiments using mRNA that were used to construct these two cDNA libraries. The results confirmed the expressions (Table 2).

**Table 2:** qRT-PCR validation of selected DEGs

Gene	Expression Change*	
	ESTs	Q-RT-PCR
HSP26	-3.8	-5.08
Cystatin B inhibitor	-7	-2.06
LEA	-7.3	-7.10
cathepsin L-like protease	-3.4	-5.29
haloacid dehalogenase-like	-1.5	-3.55
HSP70	-11	-3.57
CDC48	0/5	+3.38
chaperonin-containing TCP	+3.33	+3.66
UCP2	+2.5	+10.55
GADPH		

* Fold changes are used for expression quantization; plus and minus indicate down- and up-regulations. For the EST data, we used transcripts per million (TPM) instead of absolute expression abundance, and for Q_PCR results, we calculated fold changes directly, according to the manufacture's instruction.

## Conclusion

In this report, we described 28,039 *Artemia franciscana *ESTs and their 8,018 unigenes, representing the largest sequence resource in the public databases for this organism. We confirmed differential expression for p26, a small heat shock protein that was abundantly expressed in encysted embryos and that serves as a multifunctional molecular chaperone. Comparing to a public available dataset that derived from diapause-destined *Artemia *embryos, we noticed the critical role of p26 in both entering and maintaining dipause. We also identified two novel late embryo abundant (LEA) genes homologous to the plant LEA proteins, and together with HSP70, proteases, and protease inhibitors, these down-regulated genes after cysts reactivation provided the ability of desiccation tolerance for the encysted embryos. A similar set of genes have also been reported in plant seeds [12].

Among down-regulated DEGs, we identified 37 ribosomal protein coding genes; this is consistent with a previous finding that mRNA activity for ribosomal proteins is stored in the cytoplasm of dormant cysts and associated with polysomes [10]. Other protein coding genes may also be stored in encysted embryos. For example, a cathepsin like cysteine proteinase that was involved in yolk utilization in *Artemia *embryos [11] was enriched in dehydrated embryos. Several groups of functionally related genes were up-regulated up to 20 folds after reactivation, which include genes that are involved in energy generation, translation/transcription, metabolism, growth, cell division, and differentiation. The activation of these genes allows *Artemia *embryos to convert the stored yolk palette into energy and the result is consistent with their rapid development after reactivation.

## Methods

### Sample preparation

We obtained dehydrated *Artemia franciscana *cysts that were in the quiescence state from Dr. Jianhai Xiang's laboratory at the Institute of Oceanology, Chinese Academy of Sciences, Qingdao, China. We confirmed the taxonomic identity based on mitochondrial cox1 sequence from single cysts, a technique known as DNA Bar-coding. We evaluated the hatching rate of the cysts following a previously-described procedure [20] to optimize hatching rates. The first free-swing larva usually appear at the 10^th ^hour of hatching, 15% of the cysts hatch after 15 hours, and more than 90% of the cysts develop into free-swing larva at the 20^th ^hour. We started our experiments from dehydrated cysts and collected samples from the rehydrated cysts after 5-, 10-, and 15-hour hatching for the cDNA library construction.

### cDNA library construction and quality estimation

We extracted total RNAs from the samples using Trizol agent (Invitrogen), and isolated polyA mRNAs using PolyATtract mRNA isolation system (Promega). To obtain a broad coverage of *Artemia *transcripts, we size-fractioned double-stranded cDNAs before cloning. We constructed cDNA libraries using the directional pBluescript^® ^II XR vector (Stratagene), exploiting the EcoRI and XhoI restriction sites, according to the manufacturer's instruction. The cDNA libraries were not normalized. To assess the quality of cDNA libraries, we performed colony PCR on 96 randomly picked clones to determine the average insert size and percentage of clones without inserts and sequenced 384 randomly picked clones from each cDNA library to determine the ratio of host and vector sequences (empty clones), the sequence length after masking vector sequences, and the ratio of unique sequences (contigs + singlets)/reads.

### EST sequencing, assembly, and annotation

We acquired 5' ESTs from ~10,000 colonies of each library, using MegaBase^® ^1000 sequencers. We assembled the sequences using Phred-phrap-consed [21,22] with default parameters after removal of vector and low-quality (< 100 bp) sequences. We annotated our unigene sequences (consensus sequences of assembled clusters, including contigs and singlets) based on protein sequences in NCBI non-redundant (nr) protein database and several other public databases including *Drosophila melanogaster *protein database (dmel-all-translation; release 4.3) from FlyBase [23] and silkworm *Bombyx mori *BGF protein database database from SilkDB [24], using BLAST [25] based tools, such as blastx and tblastx. We also collected available EST sequences of Insecta and Crustacean from NCBI's dbEST. To classify the *Artemia *unigenes into Gene Ontology (GO) categories, we compared them with proteins in UniProt (uniprot_sprot and uniprot_trembl) and assigned GO terms according to their best matches, by using a UniProt2GO data provided by the European Bioinformatics Institute (EBI).

### Identification of differentially-expressed genes (DEGs) and quantitative PCR validation

We used a program IDEG6 [9,26] to identify genes that are differentially expressed among libraries. A unigene (or gene) is said to be differentially expressed when it produces a *P *< 0.05 using Chi-Square algorithm. We further validate the expression profiles of nine DEGs, using quantitative real-time PCR (qRT-PCR). We designed primers for these genes, using a program OLIGO6 with the following parameters: Tm, 60 ± 2°C, difference < = 2 between a pair of primers; primer length, 17–22 bp; GC content, 40–60%; PCR product length, 150–200 bp. Whenever possible, we chose primers that locate at the 3' end of transcripts. A full list of designed primers is shown in Table 2 and their sequences in Additional file 3.

We used the same RNA samples as what for constructing the cDNA libraries. The RNA samples were treated with RNase-free DNase I (Promega) to remove possible DNA contaminations. The first-strand cDNA was synthesized by using 500 ng total RNAs, poly(T) primers and SSII reverse polymerase (Invitrogen). qRT-PCR was conducted by using a Quant SYBR Green PCR kit (Tiangen, China). We chose the GADPH gene as an external reference for data normalization. The PCR reaction parameters were as follows: 95°C for 2 min; 40 cycles of 3-temperature of 95°C for 15 s, 60°C for 20 s, 72°C for 30 s. Three replicates for each pair of primers per template were included. qRT-PCR data were analyzed by using Opticon Monitor ^® ^software. Melting curves for each PCR were carefully analyzed to avoid non-specific amplifications. Gene expressions were quantified and transformed by using the ΔCt formula normalized with the expression of GAPDH.

## Authors' contributions

WHC, XG and WW carried out the experiments and data analysis; WHC wrote the manuscript; JY and SH designed and supervised this research and revised the manuscript.

## Supplementary Material

Additional file 1 **Differentailly expressed genes (DEGs) between cDNA libraries AfD0 and AfR5.** The data provided a complete list of differentially expressed genes we identified between AfD0 and AfR5. Click here for file

Additional file 2 **down-regulated DEGs shared between this study and published data**. We compared the down-regulated DEGs that were mostly enriched in dehydrated cysts in this study with genes enriced in diapause-destined *Artemia *embryos from a previous study (see Ref 19) and provided the gene list here. Click here for file

Additional file 3 **primer sequences used in qRT-PCR experiments.** This file provided primer sequences that we designed for qRT-PCR experiments. Click here for file
